# Transcriptomic responses to thermal and angling stress in wild brook trout from a southern Ontario stream

**DOI:** 10.1093/conphys/coaf084

**Published:** 2025-12-11

**Authors:** Andrew Howarth, Shahinur S Islam, Britney L Firth, Daniel D Heath, Steven J Cooke

**Affiliations:** Fish Ecology and Conservation Physiology Laboratory, Department of Biology and Institute of Environmental and Interdisciplinary Science, Carleton University, 1125 Colonel By Dr., Ottawa, ON K1S 5B6, Canada; Social Ecology and Conservation Collaborative, Department of Biology and Institute of Environmental and Interdisciplinary Science, Carleton University, 1125 Colonel By Dr., Ottawa, ON K1S 5B6, Canada; Great Lakes Institute for Environmental Research, University of Windsor, 401 Sunset Ave., Windsor, ON N9B 3P4, Canada; Department of Integrative Biology, University of Windsor, 401 Sunset Ave., Windsor, ON N9B 3P4, Canada; Great Lakes Institute for Environmental Research, University of Windsor, 401 Sunset Ave., Windsor, ON N9B 3P4, Canada; Great Lakes Institute for Environmental Research, University of Windsor, 401 Sunset Ave., Windsor, ON N9B 3P4, Canada; Department of Integrative Biology, University of Windsor, 401 Sunset Ave., Windsor, ON N9B 3P4, Canada; Fish Ecology and Conservation Physiology Laboratory, Department of Biology and Institute of Environmental and Interdisciplinary Science, Carleton University, 1125 Colonel By Dr., Ottawa, ON K1S 5B6, Canada

**Keywords:** Angling, climate change, exhaustive exercise, genomics, recreational fishing, thermal stress

## Abstract

Brook trout (*Salvelinus fontinalis*) are threatened by emergent and intensifying anthropogenic stressors that have uncertain cumulative effects. Effectively managing and conserving brook trout will require robust and timely information on population health—particularly where human impacts on brook trout are multiple and intense. Advanced molecular genomic tools, such as quantitative PCR assays that identify and characterize stress in fish, may provide such information, and are advancing due to an accumulation of research on transcript-level stress responses in various fishes. We used a version of the Stress Transcriptional Profiling Chip developed by the Genomic Network for Fish Identification, Stress and Health to identify changes in gene transcription related to temperature and catch-and-release angling in wild, small stream brook trout in southern Ontario’s West Credit River. We angled and took non-lethal gill tissue samples from brook trout either immediately or one hour post-capture in both cool, spring conditions and warm, midsummer conditions. Transcript abundances of *heat shock transcription factor 1* (*hsf1*), *heat shock cognate 71 kDa protein* (*hsc70*), *heat shock protein 70a* (*hsp70a*), *metallothionein A* (*mtA*), and *11β-hydroxysteroid dehydrogenase 2* (*hsd11b2*) increased significantly in thermally stressful, midsummer conditions. Transcript abundances of *hsf1* and *insulin-like growth factor 1* (*igf1*) increased after angling in cool, spring conditions, but evidence of angling effects on transcript abundances was generally weak. These results contribute to a growing understanding of transcript-level stress responses in fish, which may be used to monitor brook trout population health locally, and create tools to monitor salmonid population health more broadly.

## Introduction

Freshwater biodiversity faces an uncertain, worrisome future due to anthropogenic stressors such as land use change (e.g. urbanization, agriculture), fisheries interactions and exploitation, aquatic invasive species, pollution in various forms, and climate change (Reid *et al.*, 2019). The cumulative effects of this are severe, and have led to what is now recognized as a freshwater biodiversity crisis (Dudgeon *et al.*, 2006; Harrison *et al.*, 2018; Albert *et al.*, 2021). In Canada, vast and numerous freshwater ecosystems support diverse freshwater fisheries, which are relied upon heavily for economic, social, cultural, and other reasons (Castañeda *et al.*, 2020). Freshwater fishing in Canada is mostly recreational (Cooke and Murchie, 2013; Castañeda *et al.*, 2020), and most recreationally caught fish are released (Fisheries and Oceans Canada, 2015). Yet, recreational fishing has led to ‘invisible’ declines and collapses in some Canadian fisheries (Post *et al.*, 2002). Emerging and/or intensifying anthropogenic stressors will disproportionately impact certain freshwater ecosystems and fisheries (Chu *et al.*, 2015), but the abilities of many vulnerable species to adapt to environmental change (e.g. warming temperatures) and tolerate stress (e.g. catch-and-release angling) remain uncertain. Reducing this uncertainty will help mitigate the social, economic, cultural, and other consequences of freshwater biodiversity and fishery declines in Canada and beyond (Schindler, 2001).

Temperature is considered a critical factor in fish ecology and physiology due to its direct effect on the internal temperature, and therefore metabolism of ectotherms (Fry, 1947). Ectotherm fish (i.e. the vast majority of extant fishes) have a preferred temperature range wherein metabolism and other performance attributes are optimized, causing key activities (e.g. feeding) to be temperature driven (Brett, 1971). Outside of their preferred temperature range, ectotherm fish incur sub-lethal, and in some cases lethal stress due to their limited capacity to thermoregulate. Freshwater fish, therefore, are vulnerable to climate change (Myers *et al.*, 2017) and other related or interacting anthropogenic stressors (e.g. deforestation, recreational angling; Arthington *et al.*, 2016). These stressors include habitat degradation (leading to higher energy expenditure and/or homeostatic overload; Jeffrey *et al.*, 2015), chemical pollution (leading to various forms of physical harm; Malik *et al.*, 2020), and catch-and-release angling (leading to post-release stress or mortality; Boyd *et al.*, 2010). Each of these stressors—which form a non-exhaustive list of things that negatively affect freshwater fish health—can exacerbate thermal stress in fishes such as salmonids (González-Espinosa *et al.*, 2024).

Coldwater stenotherms like brook trout (*Salvelinus fontinalis*) are particularly vulnerable to climate change and other interacting stressors because they require cold temperatures within a narrow range, and often inhabit bodies of water wherein human activity can easily alter these conditions (e.g. small streams). Brook trout prefer temperatures of approximately 14–18°C, avoid temperatures greater than 19°C, and die as a direct result of heat at temperatures above 24°C (Cherry *et al.*, 1975; Power, 1980). Different strains of brook trout have slightly different temperature preferences, but these differences appear to diminish as acclimation temperature approaches 20°C (Stitt *et al.*, 2014). This, along with research on angling stress (Boyd *et al.*, 2010; Twardek *et al.*, 2018; Bouchard *et al.*, 2022), has led to the establishment of norms and regulations around recreational angling for salmonids in warm temperatures (Jeanson *et al.*, 2021). Short-term, temperature-related fishing closures (referred to colloquially as ‘hoot-owl’ closures) have been implemented in relatively few salmonid fisheries (Boyd *et al.*, 2010; Van Leeuwen *et al.*, 2021; Meyer *et al.*, 2023), but norms and voluntary regulations are now widespread (Hunt, 2021; Madden, 2024; Monahan, 2024; Durrant, 2025). These measures are intended to reduce post-release mortality and sub-lethal impacts that can affect whole populations and fisheries for salmonids.

As with other socially and ecologically important freshwater fishes, effectively managing and conserving brook trout will require robust and timely information on population health. Novel technologies and techniques may provide such information (Reid *et al.*, 2019), and tools like the Stress Transcriptional Profiling Chip (STP Chip) are one example of this (Semeniuk *et al.*, 2022; Islam *et al.*, 2024). The STP Chip uses qPCR to measure transcript abundances of various candidate genes that are altered in response to various general and specific stressors (Stears and Gullans, 2000), and can be combined to create various ‘fish health chips’ (Beach *et al.*, 2025; Best *et al.*, 2025; Roberts *et al.*, 2025). Candidate genes are chosen because they are involved in physiological responses to stressors, and thus indicative of fish health. For example, if abundances of RNA transcripts are found to be relatively high for a heat shock protein (HSP) involved in thermal stress responses, this indicates that sampled fish are thermally stressed. Conversely, if abundances of RNA transcripts are relatively low for a gene involved in immune responses, this may indicate that immune functions are impaired in sampled fish. Stress-related changes in transcription begin at the onset of stress, and differences in transcript abundance become detectable when stress-related genes have been transcribed at higher or lower rates for some time (e.g. for one hour). Therefore, samples taken some time after a potentially stressful event (e.g. a temperature increase, an exhaustive exercise) can reveal stress (or lack thereof) stemming from said event. Comparisons of data from samples taken immediately and after various delays (e.g. 0, 0.5, 2 h) have been used to identify stress-related changes in transcription in salmonids (Wiseman *et al.*, 2007;Donaldson *et al.*, 2014; DePasquale *et al.*, 2023) and other fishes.

Alterations of transcript abundances of stress-related genes can be detected in various tissues. Gill tissue, for example, can be used to detect stress in salmonids (Donaldson *et al.*, 2014; DePasquale *et al.*, 2023) due to the primary role of gills in homeostatic function for these and other teleost fishes (Evans *et al.*, 2005; de Oliveira Ribeiro and Narciso, 2014; Tiedke *et al.*, 2015). Insights on salmonid health are attainable even with small, non-lethal gill samples (Jeffries *et al.*, 2021), but there is still much uncertainty around transcriptomic responses to certain stressors (e.g. catch-and-release angling), in certain tissues (e.g. gill tissue), in certain species (e.g. brook trout). We used a version of the STP Chip developed by Islam *et al.* (2024) to identify transcriptomic responses to thermal and angling stress in wild, small stream brook trout. This chip included genes associated with general stress, hypoxia, immune function, growth and metabolism, and osmoregulation, as well as endogenous control genes (see Laboratory analysis). Post-release mortality and sub-lethal stress are relatively low when salmonids are angled well below thermally stressful temperatures (and not seriously injured or mishandled), and much higher when fish are angled at or near temperatures that are stressful in and of themselves (Meka and McCormick, 2005; Arlinghaus *et al.*, 2007; Boyd *et al.*, 2010; Gale *et al.*, 2013; Bouchard *et al.*, 2022; Meyer *et al.*, 2023). Based on this (and research on brook trout thermal tolerance), we hypothesized that brook trout in our study area would experience little stress when angled in spring, and significantly more stress in mid-summer—particularly after angling. Accordingly, we predicted transcript abundances of candidate genes in gill tissues from brook trout in our study area would be altered minimally by angling in cool, spring conditions, altered moderately by exposure to warm, midsummer conditions, and altered strongly by angling in warm, midsummer conditions. Our objectives were to identify transcriptomic responses to thermal stress, angling stress, and combined thermal and angling stress in southern Ontario’s West Credit River, and to evaluate the STP Chip and non-lethal gill sampling as potential monitoring tools for West Credit brook trout and similar fish populations.

### Study area

We conducted our research on the West Credit River in southern Ontario between bridges at 10th Line (43.7746°N, −80.0459°W) and Forks of the Credit Road (43.7957°N, −80.0132°W). This section of the upper Credit River has healthy coldwater habitat, and a wild population of brook trout that attracts many recreational anglers (Credit Valley Conservation, 1998). Brook trout here and in neighbouring watersheds encounter typical anthropogenic stressors like climate change, aquatic invasive species, recreational angling, and land use change (Haxton *et al.*, 2020; Smith *et al.*, 2023). Notably, municipal growth and its consequences (e.g. wastewater pollution, habitat fragmentation) have reduced the range and continuity of brook trout in the Credit River (Credit Valley Conservation, 2021). Credit River brook trout are more fragmented now than in the past (Credit Valley Conservation, 2021), and some important sub-populations are very vulnerable to ongoing development (Powers, 2021; Buckmaster, 2024). However, Credit River brook trout in our study area (and several other parts of the Credit watershed) still provide good opportunities to study wild brook trout and their responses to anthropogenic stress.

## Materials and methods

### Sampling

We took non-lethal gill tissue samples from 80 brook trout in our study area from May 17th to August 11th in 2022. All fish were angled with 2.05–2.75 m fly rods (2–4 weight), 1.36–1.81 kg line, and size 12–16 barbless hooks (per local, special regulations that require anglers to use barbless hooks and release all caught fish). Hooked fish were captured in a landing net, air-exposed for five seconds (to simulate a typical catch-and-release angling encounter), then sampled immediately (before angling-related changes in transcript abundance could be detected) or one hour post-capture (when angling-related changes in transcript abundance could be detected; Wiseman *et al.*, 2007; Donaldson *et al.*, 2014; DePasquale *et al.*, 2023). This created four treatment groups (see below) representing the baseline condition of brook trout in spring, brook trout after catch-and-release angling in spring, the baseline condition of brook trout in midsummer, and brook trout after catch-and-release angling in midsummer. Comparing these groups allowed us to assess the accuracy of our predictions that transcript abundances of candidate genes would be altered minimally by angling in spring conditions, altered moderately by exposure to midsummer conditions, and altered strongly by angling in midsummer conditions. We did not record fight time (the amount of time taken to land fish after hooking) due to the small size and relatively short fight time (<10 s) for even the largest fish in our sample. Fish that we sampled after one hour were held in black plastic containers (30 ✕ 22 ✕ 20 cm) that we anchored with rocks from the stream bed in shaded areas with moderate current speed at or immediately downstream of where the fish was hooked. We perforated the upper half of our holding containers to allow stream water to flow through while captured fish rested in the bottom half. We held no more than two fish at a time (one fish in each of our two holding containers). We snipped ~4 gill filaments of ~4 mm from the second or third gill arch of each fish, then suspended the filaments immediately in 2-ml microfuge tubes with 1.5 ml of RNA*later* solution (ThermoFisher Scientific, Waltham, Massachusetts, USA). Samples were held at 4°C for 2–8 days, then frozen at −80°C for 16–20 months.

**Figure 1:**
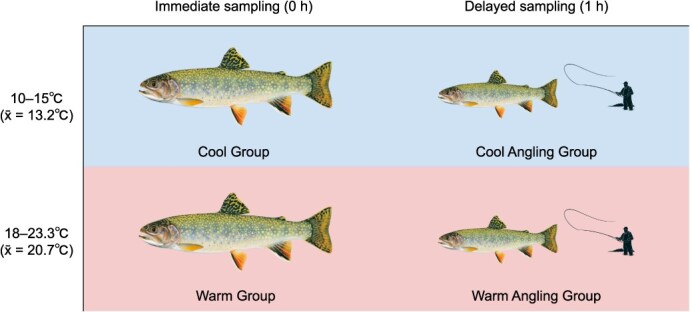
Treatment groups representing the baseline condition of brook trout (*S. fontinalis)* in cool water (reference point for changes in transcript abundance; Cool Group, *n* = 20), brook trout after catch-and-release angling in cool water (Cool Angling Group, *n* = 20), the baseline condition of brook trout in warm water (Warm Group, *n* = 20), and brook trout after catch-and-release angling in warm water (Warm Angling Group, *n* = 20). All fish were angled, but angling-related changes in transcript abundance were only anticipated in samples collected after one hour (Cool and Warm Angling Groups).

We collected our first 40 samples from May 17–20 in relatively cool water temperatures (10–15°C—non-stressful for brook trout), and our second 40 samples from August 5–11 in relatively warm water temperatures (18–23.2°C—stressful for brook trout). The latter 40 samples were from fish that we angled at or above generally agreed-upon temperature thresholds for responsible catch-and-release trout fishing (18–20°C; Hunt, 2021; Madden, 2024). The appropriateness of these thresholds has come into question (Madden, 2024; Monahan, 2024), but recommendations to abstain from trout fishing when water temperatures approach 20°C have permeated angling communities and fisheries management in recent years (Hunt, 2021; Durrant, 2025). We used a digital thermometer (TM-KIT; axGear, Blaine, Washington, USA) to record the water temperature at which fish were angled by weighting the sensor probe and measuring bottom temperature at or immediately downstream of where fish were hooked. Dissolved oxygen levels at our study site ranged from approximately 7.6–10.5 mgL^−1^ (x- = 9.0, SD = 0.8) from May 17–20, and approximately 6.7–9.1 mgL^−1^ (x- = 7.7, SD = 0.6) from August 5–11. After gill tissue sampling, we recorded the total length and clipped the adipose fin of each fish (to avoid re-sampling). Our work was approved by the Carleton University Animal Care Committee (AUP #110723) and the Ontario Ministry of Natural Resources, who granted us a Licence to Collect Fish for Scientific Purposes (Licence No. 1100288). We observed no mortalities or serious physical impairment in sampled fish. Each individual sample came from one of four treatment groups: (i) fish sampled immediately in cool water (*n* = 20, x- = 153.8 mm, SD = 25.1 mm), (ii) fish sampled after one hour in cool water (*n* = 20, x- = 153.5 mm, SD = 25.4 mm), (iii) fish sampled immediately in warm water (*n* = 20, x- = 173.2 mm, SD = 28.5 mm), and (iv) fish sampled after one hour in warm water (*n* = 20, x- = 171.7 mm, SD = 29.1 mm; Fig. 1).

### Laboratory analysis

We extracted RNA from samples (*n* = 80) using TRIzol (Invitrogen, also ThermoFisher Scientific), glass mill beads (1 mm; BioSpec Products, Bartlesville, Oklahoma, USA), and a BeadBeater (BioSpec) that we used in accordance with manufacturer directions. We assessed RNA quantity and quality using the Spark Multimode Microplate Reader (NanoQuant Plate; Tecan, Männedorf, canton of Zürich, Switzerland). This verified that our small samples yielded sufficient starting material for cDNA synthesis (≥10 ng/μl) and that RNA quality was not markedly higher or lower in any treatment group(s) (e.g. due to sampling in much warmer weather for the Warm Group and Warm Angling Group). We synthesized RNA from each sample (2 μg) into cDNA using a High-Capacity cDNA Reverse Transcription Kit (Applied Biosystems, Burlington, Ontario, Canada), then quantified and diluted cDNA for each sample to a standardized concentration (100–200 ng/μl). Synthesized cDNA samples were frozen at −80°C before qPCR. A 5 μl mixture containing 2.5 μl TaqMan® OpenArray® Real-Time PCR Master Mix (Applied Biosystems, Burlington, Ontario, Canada), 1.3 μl double distilled water, and 1.2 μl cDNA was then prepared for each cDNA sample. During qPCR, these 5 μl mixtures were prepared in 384-well plates and loaded into OpenArray® chips using the OpenArray® AccuFill System to reduce inter-assay variation. We quantified transcript abundances using a QuantStudio 12 K Flex Real-Time PCR System and 2✕ PCR Master Mix (ThermoFisher Scientific) that we used to run three custom TaqMan OpenArray chips (ThermoFisher Scientific) in accordance with manufacturer directions. In each of these runs, a technical replicate for each individual sample was produced. Our STP Chip (Table 1) included genes associated with general stress (*n* = 12), hypoxia (*n* = 4), immune function (*n* = 3), growth and metabolism (*n* = 3), and osmoregulation (*n* = 3), along with several endogenous control genes (*n* = 3). We ran this STP Chip in its entirety, but only genes with regular amplification curves and relevance to our hypotheses about thermal and angling stress were included in our analysis.

**Table 1:** Genes included in our chosen STP Chip

Category	Gene	Forward primer sequence	Reverse primer sequence	Primer efficiency (%)
Stress	*hsd11b2*	CTGTCTAGCAGCGTACGGAGC	ATGGTGGACACTTTGACCCC	83.3
	*hsd20b2*	ACATTGTACTGGTCAGCCGGT	TGGCCCTCTGTGAAGTCTGTC	94.6
	*hsp70a*	GAGAACACTGTCCTCCAGCTCC	CCCTGAAGAGGTCGGAACAC	91.5
	*hsf1*	CCCAAGTTCAGCAGGCAGTAC	GCCGTGAAGAGACCGGTACT	107.8
	*cirbp*	CGGGAAGGTCTCGTGGATT	TGGTTCTGCCATCGACAGACT	98.3
	*mmp2*	CGCTGTGGAGTTCCTGATGTT	AGGTCAGGAGAGTGGCCTAGAA	86.2
	*mtA*	TGGATCCTTGTGAATGCTCCA	CTTACAACTGGTGCATGCGC	96.4
	*mtB*	GAAAAGTTGCTGCCCCTGC	AACAGCTGGTATCGCAGGTCTT	102.9
	*hspa4*	ACTGCTGAGACCGCAATGAA	TGCGTCAGTGTAGAAGCTGGG	105.4
	*hsc70*	TAGCCAACGACCAGGGAAAC	CGTCCCCAATCAGCCTTTCT	84.3
	*serpinh1a*	AGCGCTGTGAAGTCCATCAA	TGATGATCATGGCCCCATC	83.8
	*hsp90a*	AAGATCGAGGTCACCCCTGA	GGTGCCAGACTTTGCAATGG	
Hypoxia	*hif1a*	GCTGTGGGCTGAAGAGTGATC	CTGGGTTCTCCTTCAGCTGG	
	*aldoa*	CAACGGAGAGACCACCACTCA	ACGCCACTTAGCAAAGTCAGC	96.3
	*eno1a*	TCCCTGCCTTCAACGTGATC	CCTCATGGCCTCCTTGAAGG	
	*pgk*	AGATGATCATCGGTGGTGGC	CATACAGGGAGGTGCCGATC	98.2
Immune function	*MHC-I*	AGTCCCTCCCTCAGTGTCTCTG	AGGACACCATGACTCCACTGG	93.0
	*MHC-II*	CTCACAGCAGCATCTACCCCA	CACCTGACTCTGACAGGTGCAG	
	*STAT1*	CAGAAAGGCTTCCTGGAGGG	CTCTGTGGATGTGTGGGCAT	90.7
Growth and metabolism	*igf1*	TTCAAGAGTGCGATGTGCTGT	CGCCGAAGTCAGGGTTAGG	85.5
	*igf2*	ATGTGGAGGAGAACTGGTGGAC	CCTGCTGGTTGGCCTACTGA	81.3
	*ctsd*	TTCACAGACATCGCCTGCTT	GGTACCCAGACAGACTGCCAG	99.8
Osmoregulation	*rhbg*	CACAGCCACCGTTCTGATCTC	TGATGAGGAGCTGGACAGGAC	
	*slc12a2*	CGTTTTGCATCGCTCTAGCC	AGCATAGGAGGCCAGGAAGA	
	*atp6v1ba*	AGAAGACGGCCTGTGAGTTCA	GTCCCAGCATGTCCTCAGACA	
Control	*rpl7*	TCGTCATCAGGATCAGGGGT	GAAGATCTGACGCAGACGCA	
	*ef1a*	GAAGCTTGAGGACAACCCCA	GAAGCTCTCCACACACATGGG	
	*rpl13a*	CACTGGAGAGGCTGAAGGTGT	GTGGGCTTCAGACGGACAAT	95.7

Primer efficiencies are provided for target genes that amplified correctly and were deemed relevant for our study, and for our most stable control gene (i.e. for all genes included in our analysis).

Listed genes include *11β-hydroxysteroid dehydrogenase 2* (*hsd11b2*), *20β-hydroxysteroid dehydrogenase 2* (*hsd20b2*), *HSP is 70a* (*hsp70a*), *heat shock transcription factor 1* (*hsf1*), *cold-inducible RNA binding protein* (*cirbp*), *matrix metalloproteinase 2* (*mmp2*), *metallothionein A* (*mtA*), *metallothionein B* (*mtB*), *HSP a4* (*hspa4*), *heat shock cognate 71 kDa protein* (*hsc70*), *serine proteinase inhibitor H1a* (*serpinh1a*), *HSP 90a* (*hsp90a*), *hypoxia-inducible factor 1α* (*hif1a*), *aldolase A* (*aldoa*), *enolase 1a* (*eno1a*), *phosphoglycerate kinase 1* (*pgk1*), *major histocompatibility complex class 1* (*MHC-I*), major histocompatibility complex class 2 (*MHC-II*), *signal transducer and activator of transcription 1* (*STAT1*), *insulin-like growth factor 1* (*igf1*), *insulin-like growth factor 2* (*igf2*), *cathepsin D* (*ctsd*), *Rh family B glycoprotein* (*rhbg*), *solute carrier family 12 member 2* (*slc12a2*), *ATPase H+ transporting lysosomal V1 subunit B member A* (*atp6v1ba*), *ribosomal protein L7* (*rpl7*), *elongation factor 1α* (*ef1a*), and *ribosomal protein L13a* (*rpl13a*).

### Quantitative analysis

We calculated primer efficiencies (81.3–107.8% for genes that we included in our analysis; Table 1) for our target and endogenous control genes using LinRegPCR (see Ramakers *et al.*, 2003). LinRegPCR calculates efficiency values using amplification curves for each sample and provides less variable results than other methods for analyzing amplification curves (Ruijter *et al.*, 2013; Untergasser *et al.*, 2021). We visualized our amplification data in ExpressionSuite (Software Version 1.3; Thermofisher Scientific) and removed four genes associated with general stress (*heat shock protein 90a*), hypoxia (*hypoxia inducible factor 1-alpha*, *enolase 1-alpha*), and immune function (*major histocompatibility complex class 2*) due to their irregular amplification curves. Genes associated with osmoregulation were less relevant to what we were interested in (and did not change significantly across treatments), and thus excluded from analysis. Individual samples with irregular curves (for genes included in our analysis) were also removed, but a technical replicate with an acceptable amplification curve was available in each case. This allowed us to maintain our sample size (*n* = 20) for each gene, in each treatment group. We assessed the stability of our control genes using RefFinder (see Xie *et al.*, 2023) and found that transcript abundances of all three control genes varied across treatments, though far less than our more responsive target genes (e.g. *hsp70a*, *hsf1*). We believe this was due to a major difference in mean water temperature (~7.5°C) between our cool and warm treatment groups (Fig. 1) that affected brook trout metabolism and stress strongly, and had a global effect on gene transcript abundances in our study. Ribosomal proteins (e.g. *rpl13a*, *rpl7*) and elongation factors (e.g. *ef1a*) are often used as reference genes, but they are not necessarily stable across temperatures and tissues in fish (Mahanty *et al.*, 2017; Yang *et al.*, 2022). In some cases, transcript abundances of these genes are affected by temperature (Levesque, 2016), and they may even be involved in some thermal and other stress responses (Quinn *et al.*, 2011; Wang *et al.*, 2022; Das *et al.*, 2025). Results do not suggest that control genes were involved in any stress responses in our study, but these findings from other studies support our attribution of the global effect in our study to elevated temperature.

**Figure 2:**
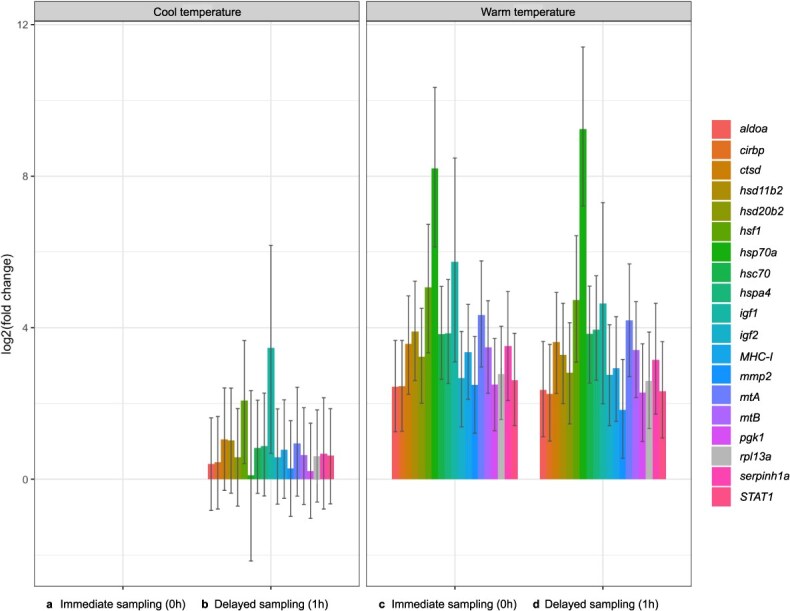
Suspected global effect of temperature on target and control gene transcript abundances in brook trout (*S. fontinalis)*. Transcript abundances of all genes are high in our (**c**) Warm Group (*n* = 20) and (**d**) Warm Angling Group (*n* = 20) relative to our (**a**) Cool Group (*n* = 20) and (**b**) Cool Angling Group (*n* = 20).

RefFinder assigned a comprehensive ranking of 1.189 to *ribosomal protein L13a* (*rpl13a*). The increase in transcript abundances of *rpl13a* in warm temperatures mirrored increases in transcript abundances of several less responsive target genes (e.g. *aldoa*, *cirbp*, *STAT1*) in our naive model (Fig. 2), and RNA quality was not markedly higher or lower in any treatment group(s). This suggested that the observed global effect was due to genes in our study being co-regulated (i.e. a true global effect), and not due to discrepancies in sampling or laboratory analysis. Accordingly, we used a modelling approach described by Matz *et al.* (2013) that provides information on the relative abundance of RNA transcripts prior to (or without) the incorporation of control gene information, and includes several workflow options for typical qPCR data analysis scenarios. This approach reformats raw qPCR data (i.e. Ct values) as molecule count data, then fits a generalized linear mixed model (GLMM) based on a Poisson-lognormal error distribution with Markov Chain Monte Carlo (MCMC) sampling. Before control genes are specified, a one-way or two-way ‘naïve’ model can be fit to explore data and reveal evidence of any global effects on RNA transcript abundances (Matz, 2013). If a global effect is present and not attributable to differences in RNA quantity and/or quality across treatments groups, a ‘soft normalization’ model that incorporates control gene information in the form of Bayesian priors can be fit to track the global effect and reveal gene-specific effects (Matz, 2013). This, and all other versions of the analysis can be implemented with the MCMC.qpcr package in R (Matz, 2013; Matz *et al.*, 2013). This package and approach to analyzing qPCR data have various advantages (e.g. joint estimation of experimental effects on all genes), and have been used in various transcriptomic studies in fish (see Velando *et al.*, 2017; Lazzarotto *et al.*, 2018; Bledsoe *et al.*, 2022; Manzon *et al.*, 2022; Guo *et al.*, 2023; Best *et al.*, 2025).

To explore our data, we fit a naive two-way model with no control gene information, sampling delay (0 h, 1 h) and temperature (cool, warm) as fixed effects, and sample ID as a random effect. This exploration revealed the aforementioned global effect (Fig. 2). Next, we fit a soft normalization model that used our most stable endogenous control gene, *rpl13a*, to normalize transcript abundances of target genes across treatments and reveal gene-specific effects. This specific approach and very similar variations of the protocol described by Matz *et al.* (2013) have proven effective in transcriptomic studies on salmonids and other fishes (Velando *et al.*, 2017; Lazzarotto *et al.*, 2018; Bledsoe *et al.*, 2022; Manzon *et al.*, 2022). We verified that our models satisfied normality and homoscedasticity assumptions by examining residuals versus predicted, scale-location, and normal quantile-quantile plots, which did not visually indicate that residuals were dependent on predicted values or distributed non-normally. Finally, we used the padj.HPDsummary function (Matz, 2013) to conduct *post hoc* pairwise comparisons with a Benjamini-Hochberg correction to identify changes in transcript abundance associated with angling in cool temperatures (Cool Angling Group compared to Cool Group), exposure to warm temperatures (Warm Group compared to Cool Group), and angling in warm temperatures (Warm Angling Group compared to Warm Group). Statistical significance was declared where Bayesian two-tailed *P*-values (*P*_MCMC_) were below 0.05.

## Results

**Figure 3:**
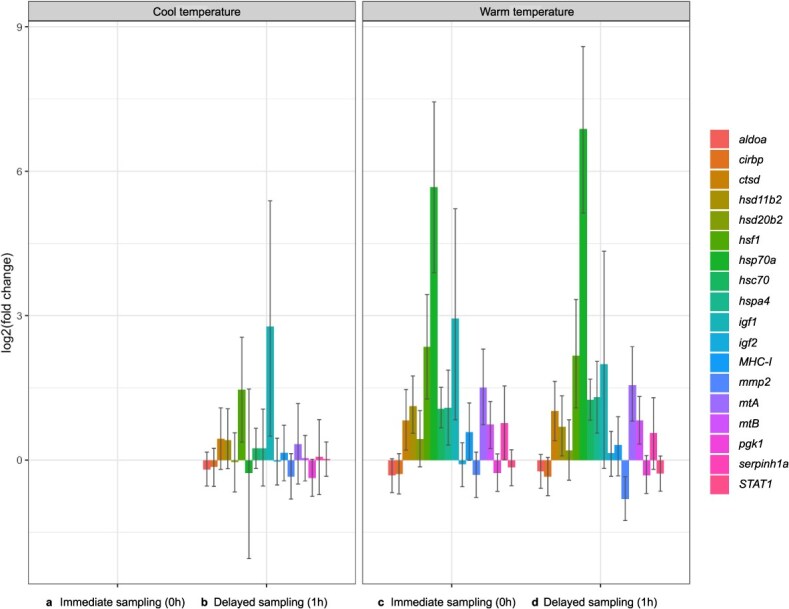
Effects of temperature and angling on target gene transcript abundances in brook trout (*S. fontinalis)* in our (**b**) Cool Angling Group (*n* = 20), (**c**) Warm Group (*n* = 20), and (**d**) Warm Angling Group (*n* = 20) relative to our (**a**) Cool Group (*n* = 20).

We included 18 target genes and one endogenous control gene in our analysis (~68% of the genes on our STP Chip), and obtained data for these genes from all 80 of our gill tissue samples, thus maintaining a sample size of 20 for all treatment groups. We found weak evidence of angling-related changes in transcript abundances for *heat shock transcription factor 1* (*hsf1*) and *insulin-like growth factor 1* (*igf1*), which increased in samples taken one hour after angling in cool water (Fig. 3). Effects of angling were significant for both *hsf1* (*P*_MCMC_ = 0.026) and *igf1* (*P*_MCMC_ = 0.042; Table 2) in the full model, but *post hoc* pairwise comparisons revealed no significant, angling-related changes in transcript abundances for either gene (*P*_MCMC_ > 0.100). Temperature-related changes in transcript abundances were found for numerous target genes in our full model (Fig. 3). Soft normalization revealed significant effects of temperature on transcript abundances of *hsf1*, *metallothionein A* (*mtA*), *metallothionein B* (*mtB*), *heat shock 70 kDa protein 4* (*hspa4*), *heat shock cognate 71 kDa protein* (*hsc70*), *igf1*, *cathepsin D* (*ctsd*), *11β-hydroxysteroid dehydrogenase 2* (*hsd11b2*), and *heat shock protein 70a* (*hsp70a*; Table 2). However, differences in transcript abundances were only significant for *hsf1* (2.4-fold increase, *P*_MCMC_ = 0.005), *mtA* (1.5-fold increase, *P*_MCMC_ = 0.014), *hsc70* (1.1-fold increase, *P*_MCMC_ < 0.001), *hsd11b2* (1.1-fold increase, *P*_MCMC_ = 0.016), and *hsp70a* (5.7-fold increase, *P*_MCMC_ < 0.001) in *post hoc* pairwise comparisons of the Warm Group (baseline condition of brook trout in warm water) to the Cool Group (baseline condition of brook trout in cool water). Interactions of angling and temperature were not significant for any genes in our full model (Table 2).

**Table 2:** Results of MCMC GLMM testing for effects of temperature and/or angling on target gene transcript abundances in brook trout (*S. fontinalis*)

Effect	Gene	Estimate	Lower 95% CI	Upper 95% CI	*P*_MCMC_	
Angling (1 h sampling delay)		0.384	−0.626	1.376	0.474	
Temperature (warm)		1.865	0.907	2.880	<0.001	***
Angling ✕ Temperature		−0.496	−1.989	0.893	0.462	
Gene-specific						
Angling	*hsf1*	1.011	0.161	1.905	0.026	*
	*mtA*	0.235	−0.539	0.865	0.476	
	*mtB*	0.029	−0.347	0.445	0.864	
	*hspa4*	0.167	−0.481	0.793	0.638	
	*hsc70*	0.171	−0.191	0.489	0.342	
	*igf1*	1.924	0.197	4.076	0.042	*
	*ctsd*	0.307	−0.231	0.827	0.236	
	*hsd11b2*	0.291	−0.259	0.789	0.258	
	*hsp70a*	−0.186	−1.742	1.335	0.814	
	…	…	…	…	…	
Temperature	*hsf1*	1.631	0.707	2.456	<0.001	***
	*mtA*	1.047	0.438	1.715	0.002	**
	*mtB*	0.514	0.105	0.889	0.008	**
	*hspa4*	0.754	0.117	1.405	0.024	*
	*hsc70*	0.739	0.380	1.086	<0.001	***
	*igf1*	2.040	0.186	3.832	0.032	*
	*ctsd*	0.569	0.044	1.109	0.036	*
	*hsd11b2*	0.781	0.289	1.293	0.004	**
	*hsp70a*	3.928	2.394	5.278	<0.001	***
	…	…	…	…	…	
Angling ✕ Temperature	*hsf1*	−1.140	−2.327	0.151	0.078	.
	*mtA*	−0.204	−1.153	0.775	0.640	
	*mtB*	0.030	−0.561	0.553	0.922	
	*hspa4*	−0.013	−0.868	0.957	0.992	
	*hsc70*	−0.040	−0.523	0.472	0.918	
	*igf1*	−2.579	−5.189	0.120	0.054	.
	*ctsd*	−0.164	−0.896	0.564	0.618	
	*hsd11b2*	−0.592	−1.268	0.103	0.120	
	*hsp70a*	1.024	−0.917	3.039	0.322	
	…	…	…	…	…	

Genes that were included in our analysis, but showed no changes in transcript abundance across experimental treatments, are excluded. Estimated coefficients are the natural logarithms of estimated fold changes.

## Discussion

Our non-lethal gill sampling technique and STP Chip yielded informative results, and our methods may be reused or adapted in various ways (e.g. by sampling with longer time delays) to provide more insight on brook trout population health in the West Credit River and elsewhere. Transcript abundances of several genes associated with cellular stress and one gene associated with metabolism increased in West Credit River brook trout exposed to warm, midsummer conditions. Angling, however, had little to no effect on transcript abundances of candidate genes in spring or midsummer conditions. Contrary to our hypothesis and predictions, alterations of candidate gene transcript abundances in West Credit brook trout were almost entirely temperature-related. On their own, these results do not suggest that stronger measures (e.g. hoot owl closures) are needed to protect West Credit brook trout from cumulative thermal and angling stress in midsummer. However, research on the effects of chronic stress on acute stress responses in salmonids (which we highlight in our Metabolism section) suggests that abstinence from angling at or near stressful temperatures is still necessary to minimize sub-lethal and lethal stress in this fish population and others like it.

### Cellular stress

Of the changes in transcript abundances that we observed across our treatment groups, increases in HSP transcript abundances in the Warm Group (baseline condition of brook trout in warm water) were most prominent. HSPs are molecular chaperones that protect cells against harmful agents including, but not limited to, high temperature (Hendrick and Hartl, 1993; Iwama *et al.*, 1998). Tissue- and time-specific upregulations of these genes occur in thermally and/or generally stressed fish (Sun *et al.*, 2021), and have been documented in salmonids, including some congenerics of brook trout (Quinn *et al.*, 2011; DePasquale *et al.*, 2023). The temperature-related 5.7-fold increase in *hsp70a* transcript abundances that we observed in the Warm Group somewhat resembles an angling-related 5-fold increase in *heat shock protein 70* (*hsp70*) transcript abundances found by DePasquale *et al.* (2023) in lake trout (*S. namaycush*). HSPs in this family have a basic cytoprotective function (Kiang and Tsokos, 1998), and upregulate quite reliably in response to thermal and/or general stress in fish (Logan and Somero, 2011; Khansari *et al.*, 2018; Sun *et al.*, 2021; Zarantoniello *et al.*, 2021). The aforementioned increase in *hsp70a* transcript abundances suggests that thermal stress resulting only from summer temperatures in West Credit River brook trout is considerable, and similar in magnitude to the angling stress found by DePasquale *et al.* (2023) in lake trout. The angling stress found by DePasquale *et al.* (2023) seemed to subside within 48 h, but the persistence of thermal stress in West Credit brook trout is unclear because we did not include treatment groups with much longer sampling delays (e.g. 12 h, 24 h).

**Figure 4:**
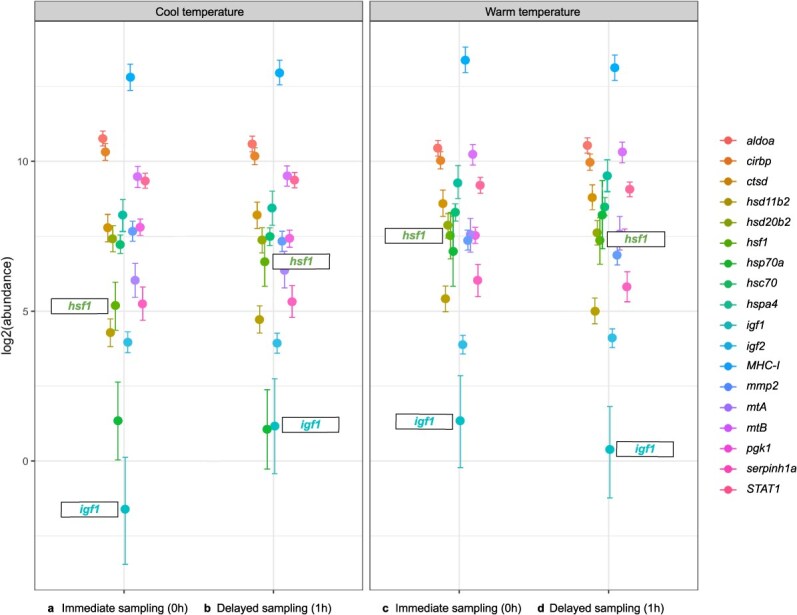
Differing effects of angling on *hsf1* and *igf1* transcript abundances in brook trout (*S. fontinalis*) in cool (left) and warm (right) temperatures. Transcript abundances of both genes are high in our (**b**) Cool Angling Group (*n* = 20) relative to our (**a**) Cool Group (*n* = 20), but not in our (**d**) Warm Angling Group (*n* = 20) relative to our (**c**) Warm Group (*n* = 20).

Expression of *hsp70* can vary substantially across individual fish and/or in field conditions, rendering it unreliable as a lone indicator of fish health and stress in some cases (Webb and Gagnon, 2009; Beemelmanns *et al.*, 2021). However, our sample size (*n* = 20) was apparently sufficient to overcome some of this variability, and we found upregulations of two additional HSPs (*hsf1*, *hsc70*) in comparisons of the Warm Group to the Cool Group. Transcript abundances increased 2.4-fold for *hsf1*, which regulates HSP transcription, and is responsive to thermal and/or general stress in fish (Buckley and Hofmann, 2002; Sun *et al.*, 2021). Transcript abundances also increased for hsc70—a molecular chaperone known to upregulate in thermally stressed salmonids (Akbarzadeh *et al.*, 2018; Shi *et al.*, 2019; Mackey, 2020). Together, increases in transcript abundances for *hsp70a*, *hsf1,* and *hsc70* suggest that typical midsummer conditions are, on their own, stressful for brook trout in the West Credit River and surrounding region. Angling could exacerbate this stress, but evidence of this was weak in our study. Effects of angling on transcript abundances may have been negligible in our study because of the small size (and short fight time) of angled fish, or because angling-related changes in transcript abundances were not detectable 1 h post-capture. Weakened stress responses in thermally stressed fish are another possible explanation for the lack of angling effects in the Warm Angling Group, but a lack of significant interactions or prominent angling effects in the Cool Angling Group do not support this. Transcript abundances of *hsf1* may have increased only after angling in cool water (Figs 3 and 4), but this alone is not strong evidence of a weakened response to angling stress in thermally stressed fish. Repeating our study with additional treatment groups and/or more delayed sampling (e.g. sampling 3 h post-capture in cool and warm water) would help evaluate these possibilities.

Increases in transcript abundances for *hsd11b2* and *mtA* in the Warm Group (compared to the Cool Group) also suggest that midsummer conditions in the West Credit River are stressful for brook trout. Increased transcript abundances of *hsd11b2*—which converts cortisol to cortisone (Mommsen *et al.*, 1999) and protects fish and their gametes from prolonged cortisol exposure (Khan *et al.*, 1997)—are yet another indication of general stress caused by ambient conditions alone. We also observed an increase in transcript abundances of *mtA*, which is very responsive to heavy metal poisoning (Hamilton and Mehrle, 1986; Dallinger *et al.*, 1997; Hansen *et al.*, 2006), but may also respond to other forms of stress (Viarengo *et al.*, 1999). Our various observations of temperature-related upregulations for genes involved in thermal and/or general stress responses suggest that brook trout are not acclimating or seeking thermal refuge effectively enough to avoid stress in warm, midsummer conditions that are increasingly typical. The severity and duration, as well as population-level effects of such stress are unclear, but it is worth noting that the West Credit River in our study area is quite accommodating to brook trout in midsummer compared to many other rivers in the region and elsewhere.

### Metabolism

Effects of temperature and angling on transcript abundances of genes associated with metabolism were minimal in our study, but we did observe some angling- and temperature-related changes in transcript abundances of *igf1* and *ctsd*. Upregulations of *igf1* are generally associated with fish growth (Wood *et al.*, 2005; Martinez-Silva *et al.*, 2023), although increases in *igf1* transcript abundances may also occur in response to certain stressors (e.g. high temperature; Zarantoniello *et al.*, 2021). Conversely, upregulations of *ctsd* correspond to upregulations of autophagy (Kantserova *et al.*, 2020) that occur, for example, in periods of starvation and growth cessation in salmonids (Cleveland and Weber, 2013; Cassidy *et al.*, 2018). Temperature seemed to affect transcript abundances of *igf1* and *ctsd* (Table 2), but these effects were not strong, and their nature was unclear. It is possible that temperature-related changes in *igf1* and *ctsd* transcript abundances were due to metabolic stress in midsummer conditions, but evidence of this was weak. It is also possible that an upregulation of *igf1* in the Cool Angling Group (compared to the Cool Group), but not the Warm Angling Group (compared to the Warm Group; Fig. 4) was due to a weakened stress response in already thermally stressed fish (as with *hsf1*). There is evidence that certain forms of chronic stress (e.g. chronic social stress, chronic thermal stress) can impair responses to more novel and/or acute stressors (e.g. heat shock, exhaustive exercise) in salmonids (LeBlanc *et al.*, 2011; Madaro *et al.*, 2015). This dampening effect of chronic stress on other stress responses may be more pronounced when these stress responses involve some of the same genes (e.g. HSPs) and may explain why, in our study, *igf1* and *hsf1* seemed to respond to angling in cool, but not warm temperatures. Evidence of a temperature-dependent response to angling was weak for both of these genes (see Results), but repeating our study with additional treatment groups (see below) might reveal effects of angling and/or temperature on brook trout metabolism and stress-responsiveness that our results only hint at.

### Tools and techniques

In addition to identifying transcriptomic responses to thermal and angling stress in brook trout, our work aimed to field-validate tools and techniques developed by GEN-FISH (Semeniuk *et al.*, 2022). Our chosen STP Chip (Table 1) yielded informative results for brook trout in our study, and shows promise as a basic and applied research tool for salmonids (Islam *et al.*, 2024; Best *et al.*, 2025). Compared to more diverse families of fishes (e.g. Cyprinidae), Salmonidae—which includes various coldwater stenotherms—may be quite conducive to the development and use of generic fish health ‘chips’. Transcript-level stress responses may vary across salmonid species, and even sexes (Donaldson *et al.*, 2014), but these responses and variations will become clearer as studies like ours accumulate. Several genes in our study *(hsf1*, *mtA*, *hsc70*, *hsd11b2*, *hsp70a*) were responsive to an elevation of temperature from cool, spring levels to warm, midsummer levels in a southern Ontario stream. Other studies like those by DePasquale *et al.* (2023) and Best *et al.* (2025) have, similarly, found certain genes (e.g. *hsp70*, *igf1*) responsive to specific stressors (e.g. catch-and-release angling, high temperature). All of these results contribute to a growing understanding of how transcript abundances relate to fish health and stress, which will allow tools like the STP Chip to be populated with reliable biomarkers (e.g. expression profiles corresponding to thermal stress).

Our study used a non-lethal gill sampling technique that proved suitable for wild, small stream brook trout. Similar non-lethal gill sampling methods have been used effectively—with negligible impacts on fish behaviour and survival—on other small salmonids, including sockeye salmon (*Oncorhynchus nerka*) smolts (Jeffries *et al.*, 2014) and juvenile lake trout (Haniford, 2023). The techniques and tools that we describe here may provide information that is currently unavailable (e.g. health status of wild, small stream brook trout) to organizations tasked with managing and conserving freshwater fish (e.g. management agencies, environmental NGOs). Among other things, this information may provide earlier indications of trouble in a fish population than some existing surveys that infer trouble from actual changes in fish abundance (e.g. a decline in population size that may take years to manifest). Our specific data also provide snapshots of brook trout health that may be used to identify changes related to future anthropogenic stress (e.g. climate warming, chemical and nutrient pollution) in the West Credit River and neighbouring systems.

Angling effects on transcript abundances were minimal in our study, which may be due to the relatively non-stressful nature of angling relatively small brook trout. Trout in small streams are often angled with barbless hooks, minimal air exposure, and very short fight times (as in our study), but angling stress may be much greater in other small stream trout fishing scenarios (e.g. due to poor fish handling). It is also possible that more delayed sampling (e.g. 2 h, 3 h post-capture) as in some other transcriptomic studies (DePasquale *et al.*, 2023) would have detected angling-related changes in transcript abundances that were hinted at in samples taken 1 h post-capture in our study. Transcript abundances of *hsf1* and *igf1* appeared to increase 1 h post-capture in cool, spring conditions, but remain the same or decrease 1 h post-capture in warm, midsummer conditions (Table 2, Fig. 3). This suggests, albeit weakly, that brook trout in our study system are thermally stressed, and less responsive to angling stress in midsummer. Sampling fish 3 h post-capture (in addition to 0 and 1 h) could determine if this is the case, or if responsibly angling and handling small brook trout (i.e. using barbless hooks, minimizing air exposure) as we did was indeed minimally stressful.

For logistic reasons, we did not include any treatment groups with much longer sampling delays (e.g. 12 h, 24 h), but doing so could answer questions about the recovery of brook trout from angling stress and/or thermal stress over hours and days. Because we did not sample fish more than 1 h post-capture, the persistence of thermal stress in West Credit brook trout is unclear. It is possible that thermal stress caused by warm temperatures in West Credit brook trout is mostly short-lived, occurring during the warmest hours of warmer days and subsiding during cooler hours from late afternoon to late morning the next day. It is also possible that thermal stress in these fish is more persistent, occurring for consecutive days or weeks of above-average temperature in the West Credit River and surrounding region. These two possible scenarios have very different implications for brook trout population health, and further research is needed to gauge the persistence of thermal stress in brook trout in the West Credit River and neighbouring systems. Our study design and application of the STP Chip were more akin to what applied scientists and practitioners—who must monitor fish populations with very limited time and other resources (Howarth *et al.*, 2023; Howarth *et al.*, 2025)—might accomplish with genomic tools like the STP Chip.

## Conclusion

Our study revealed noteworthy changes in RNA transcript abundances in wild, small stream brook trout under thermally stressful, midsummer conditions that are increasingly typical in southern Ontario and elsewhere. We found upregulations of *hsf1*, *hsc70*, *hsp70a*, *mtA,* and *hsd11b2* in fish sampled immediately post-capture in these midsummer conditions, which suggest that even high-quality habitat with relatively cool, oxygenated, and clean water (as in our study area) does not fully shield brook trout from corresponding stress. These findings underscore the importance of such habitat—which is sparser and more fragmented due to land use and development in the southern Ontario (Haxton *et al.*, 2020)—to brook trout and freshwater biodiversity in the region. Our research on West Credit River brook trout would have been difficult or impossible in many other southern Ontario watersheds where stream habitat is more degraded and brook trout are nearly or fully extirpated. Preserving research opportunities and various economic and recreational benefits currently provided by the upper Credit River will require much stronger protection of stream habitat in the West Credit River and surrounding areas. We found weak evidence of a temperature-dependent response to angling stress (i.e. upregulations of *hsf1* and *igf1* 1 h post-capture in cool, but not warm water), but more delayed sampling (e.g. 3 h, 12 h post-capture) may have been needed to assess this. Although it is possible that responses to angling stress were impaired or masked by thermal stress in our Warm Angling Group (see Discussion), we did not include treatment groups with more delayed and/or around-the-clock sampling in our study.

Follow-up studies with additional treatment groups could also answer questions about the recovery (or non-recovery) of brook trout from thermal stress across hours, days, and weeks of above-average temperature. It is possible that using best practices when angling and handling small brook trout effectively mitigates angling stress even in thermally stressful conditions, and our results do not suggest that stronger measures in fisheries management (e.g. hoot owl closures) are needed to conserve West Credit brook trout. However, the possibility that thermal stress is persistent in midsummer, or that responses to angling stress are impaired by thermal stress in West Credit brook trout, suggests that abstinence from catch-and-release angling is still the most ecologically sound course of action in extreme conditions. Cumulative evidence and remaining uncertainties in this research area suggest that anglers who choose not to catch and release salmonids at or near stressful temperatures are indeed acting in evidence-informed ways. Our work identifies several changes in RNA transcript abundances related to thermal and/or general stress in small stream brook trout during summer, which were discernible in small, non-lethal gill samples. These results may be used to assess brook trout population health in relation to further environmental change in the upper Credit River and surrounding region, and to strengthen tools like STP Chip for monitoring salmonid population health more broadly.

## Data Availability

The corresponding author will provide data associated with this project upon reasonable request.
